# Supplementary material for: Drug Cost Avoidance Resulting from Participation in Clinical Trials: A 10-Year Retrospective Analysis of Cancer Patients with Solid Tumors
Source: Cancers (Basel). 2024 Apr 17;16(8):1529. doi: 10.3390/cancers16081529 (PMC11048575; doi:10.3390/cancers16081529)
Supplement: Supplementary file 1 [file cancers-16-01529-s001.zip › cancers-2894258-supplementary.pdf]

Table S1. Pharmaceutical expenditure (€) by antineoplastic agent and study year (2010-2019) in patients treated in clinical trials.

Antineoplastic agent	2010	2011	2012	2013	2014	2015	2016	2017	2018	2019
Pembrolizumab				- €	- €	- €	€1,526,631	€2,105,884	€3,525,719	€3,590,128
Nivolumab				- €	- €	- €	- €	€2,530,787	€3,199,054	€2,489,205
Cabozantinib								€11,090	€680,013	€2,392,934
Atezolizumab			- €	- €	- €	- €	- €	- €	€1,494,348	€2,072,251
Bevacizumab	€449,063	€204,025	€318,207	€579,672	€489,726	€409,087	€477,342	€642,297	€1,023,346	€1,696,850
Palbociclib								€387,046	€1,738,509	€1,449,416
Olaparib								€1,286,924	€869,931	€1,338,767
Lorlatinib									- €	€1,094,263
Pembrolizumab/placebo						€3,657	€470,670	€645,426	€855,752	€1,076,734
Ipilimumab				€71,092	€52,203	€134,857	€96,621	€748,814	€1,380,972	€786,563
Pemetrexed	€145,919	€78,780	€179,178	€91,434	€62,875	€223,909	€309,456	€435,349	€323,262	€615,426
Pertuzumab			€22,641	€156,240	€217,308	€387,877	€490,616	€274,680	€878,976	€581,406
Avelumab						- €	- €	€172,558	€370,115	€568,416
Abiraterone				€245,325	€215,847	€454,641	€928,284	€872,218	€610,713	€500,525
Trastuzumab	€919,484	€718,598	€765,697	€615,885	€593,591	€651,436	€502,267	€401,915	€769,736	€488,859
Cetuximab	€145,079	€67,061	€169,198	€419,618	€800,388	€342,754	€211,159	€331,935	€288,909	€393,887
Lenvatinib								€307,834	€521,834	€386,417
Dinutuximab								€684,721	€822,198	€348,727
Atezolizumab/placebo							- €	- €	€237,062	€326,071
Osimertinib								€19,653	€170,345	€300,502
Sunitinib					€40,167	€90,366	€308,665	€267,347	€422,064	€299,648
Nivolumab-relatlimab/nivolumab									€88,583	€289,907
Axitinib					€100,647	€246,675	€83,063	€278,955	€266,395	€280,784
Enzalutamide							€240,697	€182,327	€240,260	€274,395

Apalutamide									- €	€259,110
Ipilimumab/placebo	- €	- €	- €	€519,158	€1,465,434	€76,700	€399,414	€550,983	€800,659	€222,315
Alecnitib									€134,477	€204,002
Panitumumab	€129,287	€107,097	€61,269	€46,033	€142,799	€100,506	€136,919	€44,676	€41,173	€183.675
Nivolumab/placebo				- €	- €	€7,127	€42,981	€146,514	€216,392	€183,521
Niraparib									- €	€181,838
Olaratumab/placebo						- €	- €	- €	€49,043	€181,812
Ribociclib								€182,270	€433,927	€178,670
Dabrafenib					€5,596	€127,578	€281,742	€175,308	€205,691	€173,907
Trastuzumab-DM1	- €	- €	- €	- €	€219,907	€1,196,442	€947,493	€1,742,676	€609,442	€168,581
Nab-Paclitaxel			€18,296	€27,430	€87,911	€235,701	€221,171	€196,538	€176,011	€164,481
Binimetinib									- €	€161,398
Ibrutinib				€55,056	€25,233		€279,320	€465,700	€170,625	€146,275
Everolimus				€749,254	€505,522	€389,669	€321,077	€249,768	€284,736	€121,903
Encorafenib									- €	€119,787
Gefitinib				€74,746	€25,024	€83,114	€11,806		€24,556	€114,629
Irinotecan pegylated liposomal			- €	- €	- €				- €	€111,976
Streptozocin						€43,620	€133,079	€185,122	€149,064	€105,370
Trifluridine-tipiracil								€116,199	€31,120	€88,155
Olaratumab	- €						- €	€40,504	€74,496	€82,608
Ceritinib									€132,222	€74,242
Talimogen laherparepvec										€66,080
Trabectedin		€221,627	€189,730	€62,459	€10,870	€14,464		€6,205		€63,041
Crizotinib					€168,476	€232,613	€132,684	€70,808	€77,750	€54,148
Cobimetinib							€93,158	€213,929	€175,978	€51,758
Regorafenib						€34,171	€213,234	€438.785	€170,585	€50,471
Vemurafenib					€183,770	€297,160	€111,461	€269,670	€229,312	€43,975

Sorafenib				€108,928	€13,612				€16,118	€38,683
Aflibercept						€3,530	€9,697	€46,737	€22,561	€37,422
Erlotinib				€305,138	€275,497	€196,250	€155,474	€80,617	€52,065	€36.678
Eribulin			€181,786	€320,152	€189,438	€65,967	€47,555	€72,440	€29,757	€33,946
Gemcitabine	€28,232	€12,981	€17,998	€28,859	€20,485	€47,946	€39,236	€21,967	€27,402	€31,796
Docetaxel	€191,287	€16,555	€5,555	€23,189	€7,789	€8,987	€2,935	€3,930	€31,434	€26,937
Letrozole									- €	€24,797
Trametinib							€137,084	€62,885	€56,818	€17,786
Cabazitaxel		€78,676	€192,252	€209,534	€66,788	€58,079	€34,728	€49,938	€18,564	€16,281
Relatlimab/Nivolumab		- €	- €			- €	- €			€15,418
Doxorubicin pegylated liposomal	€41,508	€32,668	€3,104	€47,789	€36,561	€18,494	€18,029	€25,692	€110,591	€14,328
Afatinib						€57,893	€48,572	€15,077	€71,439	€12,986
Others	€407,991	€162,072	€78,722	€420,230	€635,090	€755,820	€301,182	€699,266	€609,499	€65,460
Total (annual)	€2,457,850	€1,700,250	€2,203,633	€5,177,221	€6,658,554	€6,997,090	€9,765,502	€18,762,054	€26,011,603	€27,572,327
Total (overall)	€107,306,084									

Table S2. Pharmaceutical expenditure (€) by tumor location and study year (2010-2019), in patients treated with intravenous antineoplastic agents in clinical trials.

Tumor location	2010	2011	2012	2013	2014	2015	2016	2017	2018	2019
Lung, non-small cell	€235,003	€85,644	€322,970	€527,525	€574,879	€673,306	€1,817,333	€3,148,040	€4,096,789	€4,610,748
Advanced solid tumor	€31,986	€62,158	€111,623	€46,789	€1,038	€73,622	€129,695	€1,381,773	€1,300,154	€1,719,683
Breast	€1,139,772	€1,004,107	€972,966	€978,946	€1,298,163	€2,129,659	€2,084,398	€2,791,650	€2,683,329	€1,617,218
Colon	€443,525	€170,947	€398,663	€603,419	€946,419	€427,147	€306,086	€773,479	€1,128,681	€1,363,323
Ovary	€351,883	€142,430	€59,621	€203,510	€50,775	€20,743	€215,313	€26,356	€590,948	€1,187,763
Melanoma		- €		€140,924	€107,840	€7,127	- €	€628,359	€1,495,009	€1,053,360
Kidney	€415			- €		€78,790	€245,147	€480,786	€813,247	€889,629
Pancreas exocrine	€5,645	€5,493	€11,862	€30,493	€28,421	€236,770	€185,934	€173,100	€279,369	€502,900
Uterine cervix		€61,084,45	€6,765	€2,821		- €	€63,421	€329,780	€596,276	€461,946
Lung, small cell	€4,634	- €	€3,841	€472,254	€1,410,499	€77,120	€428,949	€1,292,808	€1,149,831	€443,215
Endometrium	€241	€682	€163	€4,350	€402	€91	€185	€5,146	€360,130	€399,268
Urinary bladder	€1,055	- €	€9,274	€41,234	€31,204	€47,944	€33,305	€58,388	€586,421	€381,720
Stomach	€8,088	€24,793	€69,671	€50,901	€113,439	€117,873	€332,654	€365,387	€634,772	€371,229
Oral cavity/oropharynx	€9,707	€51	€1,375	€24	€13,584	€91,534	€105,920	€362,824	€642,871	€353,491
Mesothelioma			€262	€110	- €	- €	€34,648	€134,060	€57,194	€287,729
Esophagus		- €		- €	€64	€6,050		€29,335	€237,976	€248,560
Soft tissue sarcoma	€267	€26,893	€75,590	€64,199	€12,081	€17,413	€6,906	€26,165	€132,348	€233,637
Rhinopharynx/cavum							- €	€130,949	€410,423	€202,101
Neuroendocrine						€43,731	€133,419	€204,239	€168,070	€126,169
Hepatic carcinoma							- €	€30,721	€16,085	€118,423
Thymoma									€14,092	€111,878

Brain		- €	- €		- €	€23,698	€14,745	€139,210	€145,677	€86,069
Prostate	€142,301	€80,071	€150,047	€155,566	€35,583	€36,306	€58,744	€105,282	€31,916	€80,384
Squamous cell skin									€72,420,00	€76,975
Larynx/hypopharynx			- €	- €	€4,672	€3,430	€8,020,	€17,082	€106,785	€59,377
Rectum	€37,596	€4,954	€322	€5,474	€5,438	€4,301	€11,304	€13,034	€31,196	€38,537
Basal cell carcinoma									€53,108	€33,102
Merkel carcinoma										€12,316
Extrahepatic bile duct	- €									€3,211
Urothelial cancer										€693
Adrenal							€10	- €		
Anus		€17,972	€7,639						- €	
Testicular germ cell		- €					- €		- €	
GIST									- €	
Osteosarcoma		- €		€3,194						
Pancreas endocrine	€45,724	€11,914	€425	€172	€56				- €	
Primary unknown					€36	€802	€287			
Ewing sarcoma		€1,051	€544		€1,976	€3,130	€3,885	€8,636	€9,620	
Thyroid							- €	€19,312	- €	
Total (annual)	€2,457,849	€1,700,250	€2,203,633	€3,331,912	€4,636,490	€4,120,596	€6,220,320	€12,675,915	€17,844,750	€17,074,664
Total (overall)	€72,266,379									

GIST: Gastrointestinal Stromal Tumors

Table S3. Mean annual cost of antineoplastic treatment per patient (€) per tumor location and study year (2010-2019), in patients treated in the Health care setting.

Tumor location	2010	2011	2012	2013	2014	2015	2016	2017	2018	2019
Adrenal							€173	€243	€4,747	€55
Advanced solid tumor	€682	€6,643	€2,706	€5,356		€707	€8,805	€2,372	€4,788	€4,316
Anus	€79	€95	€108	€139	€50	€56	€86	€840	€5,588	€2,252
Basal cell carcinoma						€69,264	€56,561	€12,888	€12,888	€25,776
Brain	€10,415	€6,032	€2,548	€3,172	€2,063	€3,707	€4,416	€2,189	€1,839	€2,421
Breast	€6,290	€5,111	€5,284	€4,847	€6,002	€6,414	€7,870	€6,938	€8,833	€10,112
Colon	€4,541	€3,086	€3,016	€1,898	€1,943	€2,725	€3,649	€3,895	€4,091	€5,475
Cutaneous tumors, other										€6,874
Endometrium	€742	€223	€182	€153	€151	€141	€123	€142	€641	€588
Esophagus	€273	€168	€197	€105	€97	€105	€89	€156	€99	€80
Ewing sarcoma	€1,273	€548	€1,239	€600	€923	€931	€2,702	€789	€2,191	€3,208
Extrahepatic bile duct	€461	€455	€511	€453	€199	€165	€337	€349	€246	€424
Gallbladder	€762			€1,177	€123	€97			€100	€78
GIST	€20,533	€28,797	€23,108	€17,643	€20,883	€21,609	€22,417	€9,338	€8,556	€4,459
Hepatic carcinoma	€12,221	€40,195	€3,404	€8,965	€6,082		€56	€3,482	€5,535	€7,673
Kaposi sarcoma	€5,307	€3,289	€1,700	€3,181	€613	€4,164	€2,240	€6,254	€634	€877
Kidney	€19,641	€20,735	€18,901	€14,380	€15,437	€23,994	€19,363	€19,043	€20,123	€21,267
Larynx/hypopharynx	€1,879	€515	€35	€2,411	€4,463	€4,968	€3,142	€4,407	€8,373	€6,837
Lung, non-small cell	€3,545	€4,428	€4,114	€3,248	€4,588	€5,730	€6,230	€9,863	€8,618	€8,371
Lung, small cell	€841	€910	€416	€168	€105	€115	€182	€240	€418	€115
Melanoma	€1,911	€928	€811	€928	€20,186	€28,412	€25,524	€24,676	€29,935	€37,020

Merkel carcinoma									€7,020	
Mesothelioma	€6,873	€7,329	€5,049	€5,995	€4,382	€4,663	€4,630	€5,204	€8,808	€85
Neuroendocrine	€24,782	€5,458	€8,550	€11,358	€11,914	€10,872	€16,484	€15,599	€13,683	€14,492
Oral cavity/oropharynx	€4,486	€2,407	€2,314	€2,902	€2,954	€3,302	€3,954	€5,695	€4,375	€5,581
Osteosarcoma	€1,568	€655	€124	€268	€573	€1,040	€1,337	€2,946	€1,127	€98
Ovary	€3,598	€1,860	€1,536	€1,457	€1,370	€2,392	€3,309	€4,859	€7,636	€5,828
Pancreas endocrine	€2,311	€6,408	€13,820	€5,013	€9,309	€17,201	€23,747	€4,475	€4,661	€134
Pancreas exocrine	€879	€627	€572	€906	€1,308	€1,712	€955	€1,263	€1,702	€2,471
Penis	€11,918	€146		€14	€343	€49	€162	€76	€140	€95
Primary unknown	€172	€177	€738	€463	€171	€1,658	€126	€304	€1,594	€8,228
Prostate	€2,992	€992	€3,303	€6,585	€9,092	€12,526	€11,195	€14,719	€12,893	€14,118
Rectum	€1,581	€1,205	€1,221	€1,408	€1,006	€1,541	€2,791	€1,570	€2,185	€2,626
Rhinopharynx/cavum	€2				€66	€72	€147	€84	€531	€115
Soft tissue sarcoma	€10,239	€4,445	€2,472	€4,500	€3,179	€4,639	€7,756	€6,545	€15,329	€7,824
Squamous cell skin										€48
Stomach	€772	€1,143	€1,012	€1,390	€903	€1,521	€1,680	€2,692	€2,962	€3,130
Testicular germ cell	€295	€233	€294	€176	€553	€1,587	€679	€133	€213	€512
Thymoma		€110	€122	€120	€52	€58	€172		€89	€219
Thyroid	€40,688	€33,975	€8,479	€6,164	€12,645	€23,027	€20,388	€14,118	€17,498	€16,256
Urinary bladder	€913	€754	€353	€318	€192	€179	€236	€48	€184	€1,319
Uterine cervix	€893	€693	€106	€60	€1,650	€859	€1,481	€1,184	€9,187	€3,968

GIST: Gastrointestinal Stromal Tumors

Table S4. Potential drug cost (€) avoided by tumor location and study year (2010-2019) in patients treated with intravenous antineoplastic agents in clinical trials.

Tumor location	2010	2011	2012	2013	2014	2015	2016	2017	2018	2019
Adrenal	- €	- €	- €	- €	- €	- €	€173	€232	- €	€27
Advanced solid tumor	- €	€14,560	- €	€643,520	- €	€5,022	- €	- €	€79,191	€45,741
Anus	- €	€216	€28	- €	- €	- €	- €	- €	€27,720	€25,030
Basal cell carcinoma	- €	- €	- €	- €	- €	- €	- €	- €	- €	- €
Brain	- €	€3,680	€1,327	- €	€3,112	€40,990	€11,218	€5,622	€11,956	€34,322
Breast	€814,295	€668,664	€652,788	€1,014,880	€1,086,657	€1,122,744	€1,321,762	€962,986	€1,375,777	€1,009,461
Colon	€468,643	€221,040	€325,021	€224,179	€246,136	€375,244	€617,870	€572,424	€609,151	€559,801
Cutaneous tumors, other	- €	- €	- €	- €	- €	- €	- €	- €	- €	- €
Endometrium	€3,150	€1,360	€660	€914	€2.254	€1,595	€545	€1,946	€28,342	€24,776
Esophagus	- €	€729	- €	€216	€204	€104	- €	€392	€1,253	€1,021
Ewing sarcoma	- €	€1,096	€222	- €	€2,864	€1,612	€1,584	€5,023	€5,967	- €
Extrahepatic bile duct	€523	- €	- €	- €	- €	- €	- €	- €	- €	€5,850
Gallbladder	- €	- €	- €	- €	- €	- €	- €	- €	- €	€78
GIST	- €	- €	- €	- €	- €	- €	- €	- €	- €	- €
Hepatic carcinoma	- €	- €	- €	- €	- €	- €	- €	- €	€470	€26,238
Kaposi sarcoma	- €	- €	- €	- €	- €	- €	- €	- €	- €	- €
Kidney	€34,896	- €	- €	€51,867	- €	€218,008	€184,695	€337,071	€583,680	€961,380
Larynx/hypopharynx	- €	- €	€36	€7,176	€21,310	€23,968	€46,640	€21,164	€78,880	€10,235
Lung, non-small cell	€115,950	€120,204	€168,492	€159,552	€282,096	€442,800	€613,900	€793,928	€1,073,257	€1,213,712
Lung, small cell	€1,052	€340	€1,490	€2,128	€872	€1,254	€5,160	€14,259	€23,562	€3,858
Melanoma	- €	€1,061	- €	€3,843	€222,957	€362,193	€362,484	€788,270	€1,404,668	€1,531,985

Merkel carcinoma	- €	- €	- €	- €	- €	- €	- €	- €	- €	- €
Mesothelioma	- €	- €	€26,190	€17,985	- €70,112	- €13,989	€64,820	€57,244	€96,888	€1.530
Neuroendocrine	- €	- €	- €	- €	- €	€2,585	€160,910	€437,502	€438,888	€235,648
Oral cavity/oropharynx	€28,311	€3,357	€14,800	€23,984	€34,276	€109,341	€289,279	€310,847	€170,380	€139,626
Osteosarcoma	- €	€826	- €	€2.256	- €	- €	- €	- €	- €	- €
Ovary	€115,841	€71.670	€77,900	€102,438	€50,320	€53,492	€80,481	€49,618	€233,044	€192,133
Pancreas endocrine	€1,002	€2,285	€3,016	€5,916	€8,378	- €	- €	- €	€272	€372
Pancreas exocrine	€5,970	€7,485	€7,530	€26,200	€46,384	€160,012	€59,706	€61,292	€76,333	€114,262
Penis	- €	- €	- €	- €	- €	- €	- €	- €	- €	- €
Primary unknown	- €	- €	- €	- €	€135	€3,328	€234	- €	- €	- €
Prostate	€26,928	€20,538	€9,703	€25,783	€36,975	€6,950	€31,092	€95,032	€79,530	€56,034
Rectum	€18,774	€9,450	€4,003	€6,412	€6,610	€6,472	€73,602	€18,224	€20,557	€23,802
Rhinopharynx/cavum	- €	- €	- €	- €	- €	- €	€248	€284	€6,895	€720
Soft tissue sarcoma	€615	€12,024	€15,270	€5,678	€11,438	€26,068	€54,768	€45,873	€315,544	€87,341
Squamous cell skin	- €	- €	- €	- €	- €	- €	- €	- €	- €	€144
Stomach	€6,942	€14,218	€12,340	€21,943	€23,303	€50,018	€82,239	€117,953	€152,280	€110,349
Testicular germ cell	- €	€249	- €	- €	- €	- €	€772	- €	€606	- €
Thymoma	- €	- €	- €	- €	- €	- €	- €	- €	€178	€1,314
Thyroid	- €	- €	- €	- €	- €	- €	€344	€572	- €	- €
Urinary bladder	€7,888	€7,272	€1,014	€2,772	€1,140	€4,186	€7,770	€997	€7,884	€39,650
Urothelial cancer	- €	- €	- €	- €	- €	- €	- €	- €	- €	- €
Uterine cervix	- €	€3,376	€565	€102	- €	€2,944	€15,323	€23,246	€363,669	€209,988
Total (annual)	€1,650,750	€1,205,680	€1,322,395	€2,349,744	€2,157,533	€3,034.919	€4,087,619	€4,722,001	€7,266,822	€6,666,428
Total (overall)	€34,463,891									

GIST: Gastrointestinal Stromal Tumors
